# Ultrasound- and Microwave-Assisted Extraction of Pectin from Apple Pomace and Its Effect on the Quality of Fruit Bars

**DOI:** 10.3390/foods12142773

**Published:** 2023-07-21

**Authors:** Angela Gurev, Tatiana Cesko, Veronica Dragancea, Aliona Ghendov-Mosanu, Adela Pintea, Rodica Sturza

**Affiliations:** 1 Faculty of Food Technology, Technical University of Moldova, 9/9 Studentilor St., MD-2045 Chisinau, Moldova; tatiana.cesko@saiem.utm.md (T.C.); veronica.dragancea@chim.utm.md (V.D.); aliona.mosanu@tpa.utm.md (A.G.-M.); rodica.sturza@chim.utm.md (R.S.); 2 Faculty of Veterinary Medicine, University of Agricultural Sciences and Veterinary Medicine, 3-5 Calea Manasturs St., 4003724 Cluj-Napoca, Romania

**Keywords:** apple pectin, ultrasound, microwave, extraction, phenolic content, antioxidant effect, biopolymer coating, dried fruits, quality

## Abstract

The article investigates the process of pectin extraction using ultrasonic and microwave techniques from apple pomace generated during juice production in the context of circular bioeconomy. The extraction yield, equivalent mass, content of methoxyl groups, content of anhydrogalacturonic acid, and degree of esterification of pectin were investigated. These indicators varied depending on the parameters and extraction method. The resulting pectin displayed a co-extracted total polyphenol content (TPC) ranging from 2.16 to 13.05 mg GAE/g DW and a DPPH radical inhibition capacity of 4.32–18.86 μmol TE/g. It was found that the antioxidant activity of raw pectin is correlated with TPC and with the content of terminal groups released during the polysaccharide degradation process. The extracted pectin was used as a binding and coating agent for dried fruit bars. Evaluation of water activity (a_w_), TPC and total flavonoid content (TFC), together with sensory and microbiological analyses of the fruit bars over a period of 360 days, revealed a protective effect of pectin: reducing moisture loss, minimizing the degradation of bioactive compounds during storage, and maintaining the potential antioxidant activity of the product.

## 1. Introduction

Apples are some of the most widespread fruits, with multiple benefits for the consumer’s health. In the Republic of Moldova, in 2017, apple production reached 430,000 tons from a total orchard area of 56,000 hectares. It was forecasted that the cultivated area for apple trees would increase by 52,400 hectares during the period of 2017–2027, resulting in a total apple production of 793,000 tons [1]. Significant amounts of apple pomace (globally, 4 million tons/year) are produced as a byproduct during the processing of apples for jams, juices, and fermented products. Despite the fact that apple pomace is primarily used as animal feed or fertilizer, it is a substantial source of functional components such as carbohydrates, dietary fibers (including pectin), phenolic compounds, and others [2]. The pectin derived from apple pomace is used in the pharmaceutical, food, cosmetic, and other industries, where it serves as a biopolymer, preservative, antioxidant, anticorrosive agent, protective agent for diverse surfaces, etc. [2,3]. Fibers obtained from fruits offer an advantage over cereal fibers due to their superior solubility, lower phytic acid content, and the presence of bioactive molecules associated with antioxidant activity [4]. Pectin is industrially obtained from apple pomace through conventional extraction (CE) methods, such as using hot acidified water with either mineral acids (sulfuric, hydrochloric, nitric) or organic acids (citric, malic, oxalic) from pH 1.5 to 3.0 and temperatures ranging from 60 to 100 °C for 0.5 to 6 h, followed by alcoholic precipitation [2,5,6]. The cost-effectiveness and optimization of pectin extraction can be improved through application of unconventional extraction techniques such as the microwave-assisted extraction (MAE) [7], ultrasound-assisted extraction (UAE) [8,9], pulsed electric field extraction [10], subcritical water extraction [11], enzyme-assisted extraction [12], as well as combinations of different extraction methods [13,14,15]. The sustainability of unconventional methods such as UAE and MAE were proved, as the methods exhibit reduced energy and reagent consumption, shorter processing times (15–30 min as opposed to 1–3 h), and improved quality and yield of the final product compared to conventional methods [16,17,18].

Several studies have confirmed that the antioxidant activity (AA) of pectin is influenced by the structure and composition of its chains, as well as by the presence of co-extracted contaminants in the polysaccharide matrix, which are associated with polyphenols, proteins, and other antioxidants [19,20,21,22]. Apple pectin, depending on its concentration, exhibited an approximately 5-fold greater DPPH radical-scavenging effect compared to other polysaccharides [23].

The pectin extraction method also influences AA. Pectin obtained by unconventional methods from various sources, with lower degree of esterification (DE) and higher anhydrogalacturonic acid (AUA) content, exhibits a higher AA compared to pectin extracted through CE [11,24,25]. Wang et al. [26] reported that pectic polysaccharides extracted with hot-compressed water from apple pomace showed in vitro AA and an inhibitory effect on free radicals. The IC_50_ values of such pectin oscillated between 1.4–3.5 mg/mL for 2,2-diphenyl-1-picrylhydrazyl (DPPH) scavenging and about 1 mg/mL for 2,2′-azino-bis(3-ethylbenzothiazoline-6-sulfonic acid) (ABTS). 

Recent studies have shown that modified pectin obtained through unconventional methods displays different structural features [26,27,28]. It contains a higher amount of galactoside residues compared to xylan and arabinan [28] and shows more advanced radical-scavenging and anticancer activities compared to native pectin and pectin extracted through conventional methods [29,30]. Hydrolytic degradation of polysaccharides during unconventional extraction is accompanied by the formation of several reducing terminal groups, respectively, by the improvement of the antioxidant potential of pectin [29,30]. 

The DE of pectin also influences its AA due to its ability to chelate heavy metal ions [31,32]. A study on the influence of modified citrus pectin on the oxidative stability of flaxseed/sunflower emulsions confirmed that low-methoxyl pectin (DE of 33%) exhibited a higher lipid antioxidant potential compared to high-methoxyl pectin (DE of 58%) [33].

Multiple research studies have shown the positive influence of pectin on health status [2,3,6,27]. Pectin has probiotic properties and contributes to the proper functioning of the intestine, as it retains water and various waste substances, facilitating the elimination of toxins and protecting the colon’s mucus membrane [31,34]. Pectin can bind and remove heavy metals from the body [31,32,34] and lower the cholesterol level [34,35] and serum glucose level [36]. Additionally, it has the capacity to capture free radicals and reduce the risk of cancer [37]. Pectic polysaccharides reduce inflammation, have antibacterial properties [38], and stimulate the immune response [39].

Pectin is used in the food industry as a thickening additive; it acts as a protective and stabilizing colloid in food and beverages. Pectin with a DE > 50% (high-methoxyl pectin—HM) forms a gel in solutions with a high concentration of sugar or solid substances, at a pH lower than 3.5. This is applied in the production of jams and jellies, fruit fillings, desserts, etc. [3]. Pectin with a DE less than 50% (low-methoxyl pectin—LM) forms a gel in a wide pH range (2.0–6.0), in the presence of calcium ions or other multivalent cations. It is used in the production of dietetic dairy products, soy-based products, etc. [40]. Other properties associated with pectin are: protein stabilization, softness in texture, increase in volume, and syneresis control in low-calorie foods [4,6,41].

Studies conducted in recent years have highlighted the sustainability of using pectin for the formulation and preservation of functional foods and encapsulation of bioactive compounds [42]. The production of edible coatings based on pectin and other biodegradable polymers is encouraged by the United States Environmental Protection Agency (US EPA) program to minimize packaging waste. Pectin-based nanoemulsions play an important role in creating a new generation of active packaging with health benefits [43]. Pectin-based films are biodegradable and possess excellent mechanical properties, providing the possibility to extend the shelf life of packaged foods [44,45], control moisture loss, and reduce the degradation of bioactive compounds during storage [46,47].

**The objective of this study** was to examine the impact of unconventional extraction methods, such as ultrasound-assisted extraction (UAE) and microwave-assisted extraction (MAE), on the yield, properties, and antioxidant activity of raw pectin extracted from apple pomace. The study also aimed to assess the potential of pectin as a binding and coating agent in the production of dried fruit bars; the protective effect of pectin films on functional products during storage was also investigated.

## 2. Materials and Methods

### 2.1. Materials

*Golden Delicious* apples were harvested in the autumn of 2021 at “AgroProduct” SRL, located in the village of Colicăuți, Briceni, Republic of Moldova (48°18′36″ N 27°8′54″ E). They were stored in a refrigerator for 10 months (Briceni) until the spring of 2022 at a temperature of 2 ± 1 °C and a relative humidity of the air of 87 ± 1%. For the production of the fruit bars, dried fruits were used: diced apples, seedless cherries, and prunes (purchased from Cazantip SRL, Chisinau, Republic of Moldova), as well as dried rosehips (purchased from Rose Line SRL, Taul, Donduseni, Republic of Moldova). The dried fruits were obtained by dehydrating fresh fruits, without any added sugar or other artificial flavors. The rosehip pulp powder was obtained from dried seedless rosehip pulp, ground to a particle size of 60 ± 10 µm.

6-hydroxy-2,5,7,8-tetramethylchromane-2-carboxylic acid (Trolox) (purity ≥ 97%) and 1,1-diphenyl-2-picrylhydrazyl-hydrate (DPPH) (≥95%) were provided by Alpha Aesar (Haverhill, MA, USA). Aluminum chloride hexahydrate (≥98%) and the standard compounds: β-carotene (≥95%), gallic acid (GA) (≥97%), rutin (≥94%), and quercetin (≥95%), were purchased from Sigma-Aldrich (St. Louis, MO, USA). Folin–Ciocâlteu phenol reagent (2.1 N) was purchased from Chem-Lab NV (Zedelgem, Belgium). Citric acid monohydrate, ethanol, *n*-hexane, methanol, diethyl ether, acetonitrile, chloroform, sodium carbonate, sodium chloride, chlorhydric acid, and sodium hydroxide were purchased from Chemapol (Prague, Czech Republic). All reagents used in this study were of analytical or chromatographic grade. All spectrophotometric determinations were performed on a UV-1900 spectrophotometer (Shimadzu, Tokyo, Japan).

### 2.2. Productions of Apple Pomace (AP)

Apple pomace was obtained after squeezing the juice from *Golden Delicious* apples. After juice extraction, the pomace was blanched in a 0.2% (anhydrous) citric acid solution for 10 min to inhibit enzyme activity and oxidative processes that could alter the properties of the pomace. The pomace was further pressed at a temperature of 25 ± 1 °C and dried using forced convection in a SLW 115 SMART laboratory oven (Pol-Eco Aparatura, Wodzisław Śląski, Poland) at a temperature of 70 ± 1 °C, until reaching a final moisture content of 12.0 ± 0.13%, and then was finely ground to a particle size of 140 ± 10 µm.

### 2.3. Characterizations of Apple Pomace Powder 

The following physicalchemical indicators of the apple pomace (AP) were determined: titratable acidity [48]; moisture and ash content [49]; fat content using Soxhlet extraction [50]; protein content [51]; total dietary fiber [52]; and insoluble dietary fiber [53]. The content of soluble substances was determined with a Kruss DR 201-95 digital refractometer (Kruss, Hamburg, Germany).

### 2.4. Methods of Extractions and Purifications of Pectin from Apple Pomace

Based on the results of preliminary attempts to obtain pectin by non-conventional UAE and MAE methods, the optimal parameters of the extraction regime were selected, conditions under which the quality and yield of pectin were superior. Samples of AP (60 ± 1 g) were placed in glass containers along with the aqueous solution of citric acid. By adjusting the acid concentration in the extraction mixture to pH 1.5, 2, and 2.5 and the liquid-to-solid ratio (LSR) to 10, 15, and 20 (*v/w*), the samples were prepared in triplicate.

The extraction of pectin using the ultrasound-assisted extraction (UAE) method (ISOLAB Laborgeräte GmbH, Germany) was performed at a frequency of 37 kHz for 15 and 30 min at a temperature of 60 ± 1 °C.

The extraction of pectin using the microwave-assisted extraction (MAE) method was performed in a microwave oven (MS23F301TAS Samsung, Zhongshan, China) with a magnetron power of 450 W for 5 and 10 min.

The extracts obtained through UAE and MAE were cooled to room temperature, subjected to centrifugation at 4000 rpm for 10 min, and the supernatants were collected. The pectin was sedimented by adding a volume of 96% ethyl alcohol, in a ratio of 1:1 (*v/v*). The suspensions were placed in a refrigerator at a temperature of 4 ± 1 °C for 12 h, after which the pectin was separated by filtration through a cotton cloth. The sediment was washed twice with a 60% ethyl alcohol solution and subjected to drying in a convection dryer (SLW 115 SMART, Pol-Eco Aparatura, Wodzisław Ślaski, Poland) at a temperature of 55 ± 1 °C until reaching a moisture content of 4.8 ± 0.1%. Finally, 54 UAE pectin samples (27 samples extracted for 15 min and 27 samples extracted for 30 min) and 54 MAE pectin samples (27 extracted for 5 min and 27 extracted for 10 min) were obtained. Subsequently, the extraction yield of pectin was calculated.

### 2.5. Pectin Characterization

The equivalent weight (EW), methoxyl group (MeO) content, anhydrogalacturonic acid (AUA) content, and degree of esterification (DE) of the extracted pectin were determined according to the methods described in the literature [54,55].

#### 2.5.1. The Preparation of Pectin Solutions for Titrimetric and Spectroscopic Analysis

First, 0.5 g of pectin was wetted with 5 mL of 96% ethanol, followed by the addition of 1.0 g of sodium chloride and carbon dioxide-free distilled water up to a volume of 50 mL. The mixture was thoroughly stirred for 10 min, then brought up to a volume of 100 mL with distilled water and left at room temperature for 30 min [54,55].

#### 2.5.2. The Determination of Equivalent Weight (EW)

After 30 min, 25 mL of the pectin solution was taken and 2–3 drops of phenolphthalein were added. This was followed by the titration with NaOH solution—0.1 N—until a constant pink color was obtained for 30 s. The EW of pectin was calculated according to the method and formula proposed by Ranaganna [54].

#### 2.5.3. The Determination of the Methoxyl Groups (MeO)

To the neutral solution mentioned above, which was titrated for equivalent mass determination, 25 mL of 0.25 M NaOH was added. The mixture was thoroughly agitated and left for 30 min at room temperature in a stoppered flask. After that, 25 mL of 0.25 M HCl solution was added to the sample, and the excess acid was titrated with 0.1 N NaOH solution until a pink color appeared. For the calculation of MeO (%), the formula in [54] was applied.

#### 2.5.4. The Determination of the Content of the Anhydrogalacturonic Acid (AUA)

The volume of NaOH (mL) consumed for MeO and EW determinations was entered into the formula for AUA calculation [55].

#### 2.5.5. The Determination of the Degree of Esterification (DE)

The degree of esterification represents the content of esterified carboxylic groups in the pectin macromolecule. The values obtained above, for MeO (%) and DE (%), were entered into the formula in [54] for calculating the AUA content. 

### 2.6. The Production of Fruit Bars

For the production of fruit bars, apples, plums, and cherries were dried until reaching a moisture content of 11 ± 1% and then crushed. For 1000 g of fruit bars, the following quantities of dried fruits were used: apples—340 g, cherries—250 g, plums—200 g, rosehip powder—6 g, and apple pectin solution—150 g, from which one-third was used as a binding agent for the fruit mixture, while the remaining two-thirds were used for coating. For the production of bars, a sample of pectin extracted through MAE for 10 min, at pH~2, in the ratio LSR 20 (*v/w*) was selected. According to the results, the MAE method ensures a higher yield (13.54%). The selected pectin had optimal characteristics required for a binding and coating agent. The aqueous solution of pectin (4.0%) with the addition of citric acid (0.04%) was prepared by dissolving the pectin in distilled water at 40 ± 1 °C with continuous stirring until complete dissolution.

The composition was molded into rectangular bars with a mass of 35 ± 1 g and dimensions of 8.5 ± 2 cm in length, 3.0 ± 0.5 cm in width, and 1.3 ± 0.5 cm in height. The bars were subjected to three rounds of glazing. After each glazing, drying was carried out at a temperature of 82 ± 2 °C for 1 h. After the cooling stage, the glossy bars were packed in polyamide/polyethylene (PA/PE) vacuum pouches and stored in the dark, at a temperature of 18 ± 2 °C, for 360 days. The sensory, physicochemical, microbiological stability, and nutritional value analyses of the fruit bars were carried out on the 1st day, 90th day, 180th day, 270th day, and 360th day of storage. 

#### 2.6.1. Sensory Analysis of Fruit Bars

The sensory analysis of the fruit bars was conducted according to [56], after every 3 months of storage, by 9 assessors using a 5-point scoring scale. The 5-point assessment system includes the following scores: 5—very good; 4—good; 3—satisfactory; 2—poor; 1—bad; and 0—very bad. Each evaluator was given a bar (35 g) and a sensory evaluation sheet with a list of descriptors. The appearance, shape, surface condition, consistency, color, smell, and taste were evaluated.

#### 2.6.2. The Physicochemical Analysis of the Fruit Bars 

During the storage period, every three months, the following physicochemical characteristics of the fruit bars were determined: pH value [57], titratable acidity [48], moisture content [58], and water activity [59]. The total polyphenols and flavonoids, as well as the DPPH antioxidant activity, were determined according to the methods described below.

#### 2.6.3. The Microbiological Analysis of the Fruit Bars 

For the microbiological analysis of the samples of fruit bars, the method for determining the count of aerobic mesophilic microorganisms and facultative anaerobes was used according to the standards in [60,61].

### 2.7. Preparation of Apple Pomace and Fruit Bar Extracts for Spectrophotometric Analysis

First, 0.5 g of sample (AP powder or ground bars) was placed into a volumetric flask and made up to a volume of 50 mL with a 50% (*v/v*) aqueous solution of ethanol. The obtained samples were extracted using the ultrasound-assisted method (ISOLAB Laborgeräte GmbH, Eschau, Germany) at a frequency of 37 kHz and a temperature of 40 ± 1 °C, for a duration of 30 min. It was followed by centrifugation at 4000 rpm for 10 min, then separation and analysis of the supernatant (extract) [62].

### 2.8. Total Polyphenols and Flavonoids by Folin–Ciocâlteu

The total polyphenol content (TPC) and flavonoid content (TFC) were determined spectrophotometrically using well-known methods [63] with some modifications [62]. 

The TPC was determined using the Folin–Ciocâlteu phenol reagent [64] in relation to a calibration curve with gallic acid standard (0–500 mg/L, R^2^ = 0.9977) and expressed in milligrams of gallic acid equivalents per 1 g of dried sample weight (mg GAE/g DW).

The TFC was determined with AlCl_3_·6H_2_O according to the quercetin (0–160 mg/L, R^2^ = 0.9972) calibration curve. The results were expressed in milligrams of quercetin equivalents per 1 g of sample DW (mg QE/g DW).

### 2.9. Total Tannins by Folin–Ciocâlteu

The total tannin content was determined by the Waterman and Mole method [65], using the Folin–Ciocâlteu reagent. The results were calculated from a calibration curve using tannic acid (0–50 mg/L, R^2^ = 0.9985) and expressed in milligrams of tannic acid equivalents per 1 g of DW of AP (mg TAE/g DW).

### 2.10. Total Carotenoids 

For the determination of carotenoids, 2 g of the sample was extracted three times with 25 mL of a solution (1:1:1; *v:v:v*, methanol: ethyl acetate: petroleum ether) using an ultrasound bath for 15 min. The filtrates were combined and analyzed spectrophotometrically according to the method described in [66,67].

### 2.11. Determination of DPPH Free Radical-Scavenging Activity

The method described by Paulpriya et al. [68] was used to determine the antioxidant activity (AA). The DPPH radical-scavenging capacity was estimated for the hydroalcoholic or aqueous solutions of samples (AP powder, pectin, or ground bars) at a concentration of 5 mg/mL. The results were expressed in µmol Trolox equivalent (TE) per 1 g of dried weight of sample (µmol TE/g DW) from a calibration curve (0–500 µmol/L, R^2^ = 0.9992) with Trolox.

### 2.12. Mathematical Modeling

To determine the influence of the pH of the ultrasonic and microwave extraction mediums on the pectin yield, the equivalent weight, the methoxyl content, the anhydrouronic acid content, the degree of esterification, the total polyphenol content, and the antioxidant activity, the MATLAB program (MathWorks, Inc., Natick, MA, USA) was used. The mutual information values, measured in bits. The more pronounced the influence of the pH of the medium in extracts on the investigated results, the higher the bit value [69].

### 2.13. The Statistical Analysis of the Results

The calculations in this research were performed in triplicate and are presented as mean values ± standard error of the mean. The Microsoft Office Excel 2007 program (Microsoft, Redmond, WA, USA) was used. One-way analysis of variance (ANOVA) according to Tukey’s test at a significance level of *p* ≤ 0.05 was carried out with Staturphics software, Centurion XVI 16.1.17 (Statgraphics Technologies, Inc., The Plains, VA, USA).

## 3. Results

### 3.1. Characterization of Apple Pomace Powder

The physicochemical properties of *Golden Delicious* apple pomace were determined (Table 1), along with the content of bioactive compounds and antioxidant activity (Table 2). The recorded values fall within the limits reported in the literature [70] and according to previous research conducted by the authors of this study [62].

The TPC in dried Golden Delicious apple pomace is 6.03 mg GAE/ g DW, and the TFC is 2.13 mg QE/g DW (Table 2). Krasnova et al. [71] determined TPC in AP obtained from different varieties of fresh apples to range from 377.10 to 740.0 mg GAE/100 g and TFC to range from 239.63 to 685.93 mg GAE/100 g AP DW.

Using the HPLC method, TPC ranging from 4.22 to 8.67 mg GAE/g and a total content of flavan-3-ols ranging from 2.27 to 9.51 mg/g DW were quantified in AP [72]. 

The concentration of tannins determined in AP at 0.61 mg TAE/g DW corresponds to the literature data, which mention a tannin content ranging from 29.11 to 73.4 mg TAE/100 g DW, depending on the variety [71]. The low content of carotenoids, 0.04 mg/g DW, is associated with the reduced amount of seeds in AP. Radenkovs et al. [73] determined a total content of carotenoids in the recovered oil from AP ranging from 14.5 to 5.1 mg/mL. The DPPH antioxidant activity of AP, at 22.82 µmol TE/g DW, has values consistent with those recorded in the previous study (2.43 mmol TE/100 g DW) [62] but also those by Gorjanović et al. [74] (between 2.2 and 4.5 mmol TEA/100 g AP DW). 

### 3.2. Characterization of Pectin from Apple Pomace

#### 3.2.1. Pectin Yield (PY)

The conducted research has shown that the yield of pectin from Golden Delicious apple pomace depends on the applied extraction method, whether it is UAE or MAE. The PY increases as the pH decreases, the extraction time duration increases, and the liquid-to-solid ratio (LSR) (*v/w*) increases, as shown in Table 3.

Through the MAE method, pectin was extracted with a higher yield ranging from the minimum value of 1.18% (pH of 2.5, LSR of 10 (*v/w*), and 5 min extraction time) to the maximum value of 19.88% (pH~1.5, LSR of 20 (*v/w*), for 10 min), compared to the UAE pectin yield, with values between 0.98% (pH~2.5, LSR de 10 (*v/w*), for 15 min) and 9.91% (pH~1.5, LSR of 20 (*v/w*), for 30 min). The obtained results fall within the range described in the literature [75,76,77,78]. The pectin extracted from *Malus domestica* “Falticeni” apple pomace, using UAE, exhibited a yield ranging from a minimum of 1.68% (60% amplitude, pH of 2.5, LSR of 20 (*v/w*), and 20 min extraction time) to a maximum of 9.18% (60% amplitude, pH of 1.5, LSR of 15 (*v/w*), for 30 min) [75].

Multiple studies have confirmed that the MAE method yields higher extraction efficiency compared to CE and UAE [12,76,77]. Dranca et al. [78] extracted pectin from apple pomace using citric acid. At the same LSR, higher PYs were obtained using the CE method (23.26%) and the MAE method (23.32%), compared to UAE (9.18%).

Calvete et al. [12] extracted pectin from AP using the UAE and CE methods, achieving PYs of 7% and 10%, respectively. Wang et al. [16] determined the optimal MAE conditions for extracting pectin from apple pomace with a PY of 23%: extraction time of 20.8 min, pH~1.01, LSR of 14.5 (*v/w*), and power of 499.4 W. Pectin extracted using citric acid through the conventional method by Rascón et al. [79], from low-quality Golden Delicious apples, had a yield of 16%.

Based on our research, the PY appears to be quite good, considering that the apple pomace was obtained from low-firmness apples stored for 10 months. Under these conditions, depolymerization, degradation, and solubilization of biopolymers, including protopectin, take place [80,81]. The MAE method provided a PY that was twice as high compared to UAE. The phenomenon is due to the influence of microwave radiation on polar water molecules, which rapidly disperses the generated thermal energy throughout the extraction mixture, creating conditions for advanced hydrolysis of protopectin and thus increasing the yield of the final product [16,80,82].

#### 3.2.2. Equivalent Weight (EW)

According to the results illustrated in Table 3, the EW of pectin extracted through UAE and MAE decreases as the pH decreases, and the extraction time increases. The pectin with the lowest EW was obtained at a pH of approximately 1.5 in both techniques. Due to more advanced macromolecule degradation caused by ultrasound, EW of pectin extracted through UAE was slightly lower compared to MAE. The UAE pectin had a minimum EW of 378.3 g/mol (pH~1.5, LSR of 20 (*v/w*), for 30 min) and a maximum of 1927.1 g/mol (pH~2.5, LSR of 15 (*v/w*), 15 min). The MAE pectin had a minimum EW of 421.6 g/mol (pH~1.5, LSR of 20 (*v/w*), 10 min) and a maximum of 2261.7 g/mol (pH~2.5, LSR of 20 (*v/w*), 5 min), as shown in Table 3. 

The obtained data align with the information described in the literature. Dranca et al. [75] recorded an EW of 704 g/mol for UAE pectin extracted from *Malus domestica* apple pomace (at 20 kHz, pH~1.8, for 30 min, LSR of 10 (*v/w*)). For the MAE pectin, they obtained an EW of 1612 g/mol (560 W, pH~2.2, 120 s, LSR 10 (*v/w*)). The CE-extracted pectin from *Malus pumila* pomace, using HCl and citric acid (at pH~2.5, 97 °C, for 30 min), exhibited different EWs, with values of 833.33 and 1666.30 g/mol, respectively [35].

According to the literature, the EW values of pectin extracted from immature fruits are higher compared to those extracted from ripe or long-stored fruits [80,83].

In our research, the equivalent mass of pectin is likely to be lower due to the quality of the raw material used. At the same time, varying the extraction parameters of UAE and MAE allows for obtaining pectin with different EWs.

#### 3.2.3. Methoxyl Content (MeO)

The MeO content in pectin extracted from *Golden Delicious* apple pomace depends on the applied method of extraction. The results in Table 3 show that the MeO concentration declines with decreasing pH and increasing extraction time, while it is less reliant on LSR.

In MAE, pectin with a minimum MeO content of 4.88% (pH~1.5, LSR of 20 (*v/w*), for 10 min) was obtained and a maximum of 6.39% (pH~2.5, LSR of 20 (*v/w*), for 5 min). In UAE, the lowest MeO content was of 5.05% (pH~1.5, LSR of 20 (*v/w*), 30 min) and the highest was of 6.81% (pH~2.5, LSR of 20 (*v/w*), 15 min) (Table 3). 

The obtained results are comparable to those recorded by other researchers. It has been reported that in sweet potato pectin, increasing the sonication power from 100 W to 400 W reduces the degree of methoxylation from 12.0% (in native pectin) to between 5.25% and 6.28% [25]. The pectin extracted through CE from *Malus pumila* pomace and *Spondias dulcis* had methoxyl group concentrations of 6.21% and 5.68%, respectively [35]. 

In other studies, it has been demonstrated that the degree of methoxylation of pectin depends not only on the extraction method and conditions but also on the source of the pectin. As fruits ripen and during storage, the content of MeO groups in pectin decreases. The content of MeO groups in pectin extracted from mature lime pomace (4.24%) and ripe lime pomace (4.26%) was significantly lower compared to that of pectin extracted from immature lime pomace (10.27%) [83].

The results obtained in the research indicate that pectin obtained through unconventional methods such as UAE and MAE has a methoxylation degree of less than 7% and can be applied in the food industry.

#### 3.2.4. Anhydrogalacturonic Acid (AUA) Content 

The purity of the extracted pectin is determined by the content of galacturonic acid, which should be higher than 65% [84]. The research results (Table 3) have shown that the AUA content in pectin obtained through UAE and MAE methods varies from sample to sample, depending on the extraction conditions, and increases with decreasing pH, increasing LSR, and longer extraction times, as shown in Table 3.

The UAE pectin had a higher purity compared to MAE. In the UAE method, the minimum AUA content was 49.16% (pH~2.5, LSR of 15 (*v/w*), for 15 min) and the maximum was 78.71% (pH~1.5, LSR of 20 (*v/w*), for 30 min). The MAE pectin had AUA content of a minimum of 47.31% (pH~2.5, LSR of 10 (*v/w*), 5 min) and a maximum of 73.02% (pH~1.5, LSR of 20 (*v/w*), 10 min). 

Multiple research studies have demonstrated that the application of unconventional techniques such as UAE and MAE allow for obtaining a purer pectin with a higher AUA content [25,32,72]. Calvete et al. [12] extracted pectin from pomace of nine apple varieties using the CE and UAE methods. The CE pectin had an AUA content ranging from 18% to 67%, while the UAE pectin had an AUA content ranging from 48% to 75.4%. Dranca et al. [78] obtained pectin with increased AUA content from the pomace of *Malus domestica* using the CE method (86.5%) and unconventional methods such as UAE (92.83%) and MAE (90.6%). According to the literature, pectin extracted through CE, from low-quality Golden Delicious apples, had an AUA content of 65% [79].

#### 3.2.5. Degree of Esterification (DE)

The DE determined for pectin extracted from Golden Delicious apple pomace, using UAE (37 Hz), is slightly higher, with minimum and maximum values ranging from 36.47% (pH~1.5, LSR of 20 (*v/w*), for 30 min) to 73.78% (pH~2.5, LSR of 15 (*v/w*), for 15 min), compared to MAE, with values ranging from 38.69% (pH~1.5, LSR of 20 (*v/w*), 10 min) to 71.37% (pH~2.5, LSR of 10 (*v/w*), 5 min). In both methods, DE decreases with decreasing pH and increasing extraction time and is less dependent on the LSR (Table 3). These data are consistent with previous research showing that pectin extracted from AP, using conventional methods, has a higher DE (84.4%) compared to UAE (77%) and MAE (73.8%) [78]. Several studies have reported a decrease in DE of pectin extracted using non-conventional methods compared to CE [85,86]. At the same time, other researchers state that non-conventional methods give pectin with a higher DE [44,76] compared to CE pectin. Through CE, apple pomace was obtained with various DEs: 45.98 and 52.51% [35], 68.84% [87], and pectin from low-quality Golden Delicious apples with a DE of 57% [79].

Hosseini et al. [88] reported that the DE of pectin extracted from citrus peels decreases with decreasing pH, increasing power, and longer exposure to microwave radiation. Under these conditions, more advanced de-esterification of the carboxyl groups takes place. The pectin extracted from overripe lemon pomace exhibited a lower DE (33.59%), compared to the pomace from mature fruits (70.39%) and premature fruits (79.51%) [83]. DE of pectin also decreases with the duration of fruit storage [80,81].

The data presented in Table 3 show that the extraction conditions with the highest PY (19.88%) and the highest AUA content (73.02%) resulted in pectin with a DE below 50% (38.69%). The pectin DE in the present study is lower due to the applied extraction methods and the quality of the pomace obtained from Golden Delicious apples with reduced firmness after 10 months of storage (at the end of a season).

#### 3.2.6. The Antioxidant Activity (AA) of Pectin

One of the objectives of the research was to study the AA of raw pectin extracted using UAE and MAE from the pomace of Golden Delicious apples. In this context, the total polyphenol content (TPC) was determined in the samples of pectin, and the DPPH radical-scavenging capacity was estimated, as shown in Table 3.

The AA of pectin, according to the bibliographic data, depends on the source of pectin, extraction method, content of non-pectin impurities (such as polyphenols, proteins) [19,20], equivalent weight, structure and composition, degree of esterification, etc. [23,30]. Polyphenols present in AP (quercetin glycosides, phloridzin, phloretin, epicatechin, chlorogenic acid, etc.) are largely responsible for the antioxidant effects [89] and can be co-extracted in the matrix of raw pectin.

The experimental data recorded in Table 3 indicate that TPC and AA of pectin are less dependent on the liquid-to-solid ratio (*v/w*) at the same extraction parameters. As observed, the TPC of raw pectin decreases with decreasing pH and increasing extraction time. In UAE pectin, the minimum TPC was 2.16 mg GAE/g DW (0.22%) and the maximum was 12.98 mg GAE/g DW (1.30%). In MAE pectin, the minimum TPC was 2.28 mg GAE/g DW (0.23%) and the maximum was 13.05 mg GAE/g DW (1.31%). It has been noted that in the complex matrix of high molecular weight pectin macromolecules, more phenolic antioxidants are retained, as shown in Table 3.

Smirnov et al. [20] reported that fruit pectin fractions isolated from fruits, using a simulated gastric fluid, had a TPC of 0.5–0.7%. Wikiera et al. [19] obtained pectin from apple pomace by CE with a lower TPC of 0.71% compared to enzymatically extracted pectin, which had TPC ranging from 0.98 to 1.34%.

**Figure 1:**
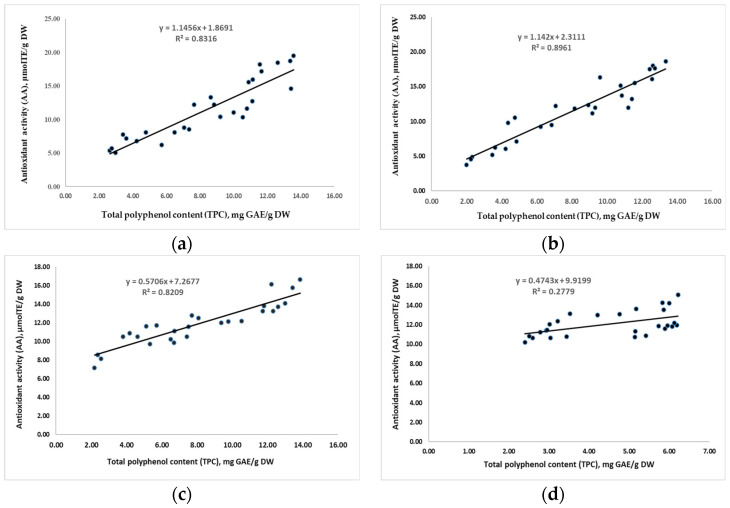
Correlation between total polyphenol content (TPC) and DPPH antioxidant activity (AA) of pectin: (**a**) Ultrasound-assisted extraction, 15 min; (**b**) ultrasound-assisted extraction, 30 min; (**c**) microwave-assisted extraction, 5 min; (**d**) microwave-assisted extraction, 10 min.

The antioxidant activity of pectin obtained through UAE and MAE evolves differently. The DPPH radical-scavenging effect of pectin extracted at the same ultrasound frequency for 15 and 30 min increased proportionally (R^2^ = 0.8316 and R^2^ = 0.8961) to the content of phenols, which are responsible for the antioxidant effect (Figure 1a,b). The DPPH inhibitory capacity of UAE pectin ranges from 11.17% to 30.74%, with values ranging from 4.32 to 18.86 μmol TE/g DW, as shown in Table 3.

The bibliographic data show that apple pectin (0.5 mg/mL) inhibits DPPH by approximately 25% [23]. Wang et al. [11] reported that pectic polysaccharides extracted from AP with hot-compressed water showed in vitro AA, and the IC_50_ values of such pectin oscillated from 1.4–3.5 mg/mL for DPPH scavenging.

In the MAE technique (at the same power of 450 W), the AA of pectin extracted for 5 min increased proportionally with the concentration of polyphenols (R^2^ = 0.8209), as shown in Figure 1c. The DPPH inhibition capacity ranged from 7.85 to 16.39 μmol TE/g DW (corresponding to 18.21–31.07%). AA of MAE pectin, extracted for 10 min, did not increase proportionally with TPC (R^2^ = 0.2779), as shown in Figure 1d. Despite the lower concentration of phenolic antioxidants in all samples, the DPPH inhibition capacity of the pectin did not significantly decrease, ranging from 10.43 to 14.68 μmol TE/g (Table 3).

Based on the recorded results, we can conclude that AA of pectin is largely associated with TPC. In the case of MAE pectins extracted for 10 min, the dependence of AA on TPC is insignificant, with this regime being destructive for polyphenols. Probably, the AA of these pectins is associated with a higher AUA content, with lower DE and lower EW, as shown in Table 3. Additionally, the increased AA of pectin extracted under prolonged microwave treatment can also be associated with the release of reducing terminal groups during the degradation process of polysaccharides. Several studies have established that the degradation and modification of pectin under the action of ultrasound or microwaves are accompanied by an increase in its antioxidant potential [13,15,24,30,35]. 

The results of the conducted research have shown that the green methods UAE and MAE are easily controllable and can be applied to obtain pectin with predictable properties. 

### 3.3. Mathematical Modeling

The mutual analysis of information was applied to determine the influence of pH (1.5, 2, 2.5) on PY, ME, MeO, AUA, DE, TPC, and AA of pectin obtained by UAE and MAE at all LSRs of 10, 15, 20 (*v/w*), as shown in Table 4. 

Data from Table 4 show that in UAE, for 15 and 30 min, pH significantly influences pectin ME (mutual information 0.998 bits), DE (0.995 and 0.996 bits), and MeO concentration (0.958 and 0.836 bits). With decreasing pH and increasing extraction time, ME, degree of methoxylation, and DE decrease. The influences of pH on PY (0.885 and 0.873 bits) and AUA content (0.836 and 0.985 bits) follow in descending order, respectively. PY and AUA increase slightly with decreasing pH, as shown in Table 3.

In the microwave extraction for 5 and 10 min at all LSRs, the PY decreases proportionally with the increase in the pH of the medium (0.998 bits), the ME decreases with the decrease in pH (0.996 and 0.982 bits, respectively), and the MeO concentration is less influenced by pH change (0.755 bits), as shown in Table 4. When microwaved for 5 min, pH has a higher influence on DE (0.996 bits) and TPC (0.916 bits) and less influence on AUA (0.821 bits) concentration and AA (0.325 bits) of pectin.

In both types of techniques (UAE and MAE), the lowest TPC was recorded at pH 1.5 in the extraction medium. In the case of a shorter time of ultrasound action (15 min), TPC values do not change much at pH 2 and 2.5 (0.491 bits). Extending the ultrasound action time to 30 min shows a significant influence of pH on AA (0.915 bits) and less on TPC (0.812 bits). 

The results of the mathematical analysis (Table 4) show that when extending the microwave action time to 10 min, a reduced influence of pH on TPC (0.522 bits), as well as an insignificant influence on AA of pectin (0.101 bits), is observed. In these conditions, TPC has low values in all samples and, at the same time, AA does not decrease, as shown in Table 3.

Mutual information analysis was applied in researching the influence of different salt concentrations and different pH values on the color parameters and antioxidant activity of rosehip extracts [90]. The same mathematical modeling procedure was used to elucidate the influence of extraction temperature on the content of biologically active compounds in grape pomace extracts [91].

### 3.4. The Characteristics of Fruit Bars

#### 3.4.1. Sensory Assessment

The sensory characteristics (appearance, shape, surface condition, consistency, color, taste, and smell) of the fruit bar samples were analyzed on the first day and every three months, until the end of the 360 days of storage. It was demonstrated that the use of pectin as a binding and coating agent positively influenced the external appearance, consistency, color, and aroma of the bars, which were preserved for 360 days (Table 5). It was established that during storage, the sensory characteristics of the bars did not change. Each descriptor was rated with up to 5 points by evaluators. Additionally, it was observed that at the end of the storage period, the cherry fruit flavor became more pronounced. These results could also be influenced by the properties of the pectin used, which was obtained under conditions (MAE for 10 min, pH~2) that led to an increase in the AUA content (65.68%), a decrease in DE (47.74%), and a reduction of EW (596.4 g/mol); properties associated with a higher AA (13.36% µmol TE/g DW) of pectin. At the same time, a pH of ~2 in the extraction medium is less destructive for pectin polyphenols (TPC 4.97 mg GAE/g DW).

#### 3.4.2. Evolution of Physicochemical Parameters and Microbiological Stability of Fruit Bars during Storage

During the storage of the bars, the following physicochemical quality parameters were analyzed (moisture content, pH, titratable acidity, and water activity), as shown in Table 6. It was shown that during storage, the moisture content of the bar samples gradually decreased from 30.00% (on the first day) to 23.6% (360th day), i.e., by 21.3%.

The pectin protective film on the surface of the bars acted as a barrier in controlling moisture retention, reducing the interaction processes between food molecules and the surrounding environment, and decreasing gas exchange. According to the literature, the moisture loss during the storage of dried plums can vary between 16.5% and 24%, depending on the coating layer of the plums [92]. 

The use of various fruits in the formulation of fruit bars results in a slightly acidic pH, which is associated by consumers with the taste of dried plums and sour cherries. The pH evolution in the bars during storage is influenced by the acidic environment formed in the dried fruits [93], as well as the use of citric acid in the preparation of the pectin solution. Over the course of 3 months, the pH value remained constant at 3.61. A slight increase in pH is observed from 3.64 (on the 180th day) to 3.95 (on the 360th day), as shown in Table 6. The titratable acidity decreases during the storage of the samples from 1.12% (on the first day) to 0.83% expressed as citric acid (360th day), due to the physicochemical transformations of compounds that occur in the fruit bars. The specialized literature confirms that the moisture content in dried fruits is approximately 20%, and the pH varies from 3.1 to 4.0, making them foods with high acidity [94].

The a_w_ determined in the bars over a period of 360 days changed from 0.571 to 0.496, indicating a decrease of 14%, as shown in Table 6. The values of water activity demonstrate the proper preservation of the fruit bars, as well as the protective and stabilizing effect of pectin as a binding and coating agent. Specialized literature confirms that the water activity in fruit bars falls within the range of 0.4 to 0.6, which is typical for dried fruits [93]. It is mentioned that dried cherries have the following physicochemical parameters: average moisture content ranging from 9.5% to 12.1%, a_w_ ranging from 0.54 to 0.66, total acidity (mEq/100 g) ranging from 2.4 to 4.43, and pH ranging from 3.8 to 4.1 [95]. Arendase et al. [94] established that the a_w_ in dried apples ranged from 0.3 to 0.4. These values negatively influence the development of molds and yeasts. The pretreatment of apples with a 0.2% citric acid solution during blanching promotes both the preservation of a light yellow color and the creation of an acidic environment to inhibit the growth of pathogenic microorganisms. From a microbiological standpoint, the reduction in moisture content, active acidity, protective layer of pectin solution, and vacuum packaging have halted the growth of microorganisms during storage, ensuring microbiological stability.

#### 3.4.3. The Biologically Active Compounds and Antioxidant Activity (AA) in Fruit Bars

The analysis of bioactive substances in fruit bars has demonstrated that the inclusion of dried fruits in the composition of the bars, along with the use of raw pectin as a binding and coating agent, has a positive influence on the evolution of antioxidant content during storage. The phenolic and flavonoid content, in the first six months of storage, remained close to the initial values, with TPC at 7.68 mg GAE/g DW and TFC at 2.75 mg EQ/g DW. However, towards the end of the storage period, the contents decreased to 5.59 mg GAE/g DW and 1.85 mg EQ/g DW, respectively. Pectin plays a stabilizing role, serving as a protective barrier, and contributes to reducing the degradation process of bioactive compounds during storage. 

According to the literature data, the TPC and TFC of the fruit bars fall within the range of values determined for dried fruits such as apples, sour cherries, plums, and rosehips. The Granny Smith, Royal, and Fuji apple varieties, when dried for 4 h at 60 °C, had TPC values of 372.81 mg GAE/100 g DW, 391.47 mg GAE/100 g DW, and 358.54 mg GAE/100 g DW, respectively [96].

In fresh cherries, TPC was determined to be 145.77 mg GAE/100 g FW, and TFC was found to be 6.49 mg GAE/100 g FW [97]. In dried sour cherries, TPC varied between 1539 and 2982.51 mg/100 g DW, depending on the variety, and total flavonoids accounted for approximately 40% of the total polyphenols [98]. Prvulovic et al. [99] measured the concentrations of total polyphenols (in a range from 4.12 to 8.34 mg GAE/g DW), flavonoids (0.42–1.56 mg of rutin equivalents/g DW), and total anthocyanin (0.35–0.69 mg cyanidin 3-glucoside equivalent/g DW) in fruits of sweet cherry genotypes. 

In ripe plums of the Qiangcuili and Cuihongli varieties, TPC was measured to be 2.20 and 2.18 mg GAE/g FW, respectively [100]. In dried plums [101], TPC reached 636.1 mg GAE/100 g, and TFC was 213.5 mg/100 g DW of plums.

According to Japanese researchers [102], the TPC in dried skinless apples was found to be 916 mg GAE/100 g, in dried cherries—828 mg GAE/100 g DW, and in dried plums—1032 mg GAE/100 g DW.

TPC of rosehips ranged from 5.77 to 10.30 g GAE/100 g [103]. However, other authors have reported lower TPC values in ethanolic extracts of rosehips, ranging from 255.9 mg to 766.0 mg GAE/100 g DW [104].

The AA in the fruit bars initially had high values of 24.85 µmol TE/g DW, which slightly decreased during the first 6 months and reached 20.14 µmol TE/g DW at the end of 1 year of storage, as shown in Table 6. These values did not undergo significant changes during the storage period due to the chemical composition of the vegetable matter and the properties of the pectin used in the manufacturing of the bars.

The literature study confirms the increased values of AA determined in dried fruits. The antioxidant capacity of dried apples was determined to be 7.62, 5.1, and 4.24 mmol Fe/100 g DW, depending on the variety. The treatment of apples with citric acid and sodium bisulfite before drying had a protective role for polyphenols [96]. In dried sour cherries, an AA of 692.9 mg TE/100 g FW, which corresponds to 2.52 mmol TE/100 g, was determined [97]. In the ethanolic extract of 70% water content from ripe plums, using the ultrasound-assisted method (40 Hz) for 15 and 30 min, Nowak et al. [105] determined a DPPH-scavenging activity of 269 and 314 mg TE/100 g FW (or 1.07 and 1.25 mmol TE/100 g FW), respectively. In two varieties of plums [101], the DPPH activity was determined, which depended on the concentration of TPC and ranged from 2.5 to 13.0 μmol TE/g FW.

The present study has shown that raw pectin containing polyphenols, modified through ultrasound or microwave treatment, and subsequently used in the formulation of bars as a binding and coating agent, maintained the AA and high functional value of the product over a storage period of 12 months.

## 4. Conclusions

The research results demonstrated that the non-conventional methods of UAE and MAE represent sustainable and easily controllable processes for obtaining pectin with anticipated properties for various applications.

Pectin obtained from Golden Delicious apple pomace showed different EW, MeO, AUA and DE values, depending on the extraction conditions. The MAE method provided a maximum pectin extraction efficiency of 19.88%, while the UAE method—9.91%.

The TPC in the raw pectin matrix obtained by non-conventional methods varied from 0.22–1.31%. The DPPH radical inhibition capacity of pectin aqueous solution showed values from 4.32 to 18.86 μmol TE/g DW, this being largely dependent on the TPC. The AA of pectins obtained by MAE for 10 min were associated with a higher content of AUA, with a lower DE and EW, as well as with the content of terminal reducing groups, released during the polysaccharide degradation process. MAE pectin, selected for bar production, had the optimal characteristics required for a binding and coating agent. The evaluation of the physicochemical and sensory parameters of the fruit bars every 3 months, over a period of 12 months, demonstrated the protective effect of pectin: reducing moisture loss, minimizing the degradation of bioactive compounds during storage, and maintaining the potential antioxidant activity of the product.

## Figures and Tables

**Table 1:** Physicochemical parameters of the apple pomace powder.

Parameters	Mean Value ± SD
Moisture content, %	12.0 ± 0.13
Titratable acidity, % expressed in malic acid	0.19 ± 0.01
Soluble solids content, o Brix	15.54 ± 0.03
Fat content, %	2.74 ± 0.21
Protein content, %	4.67 ± 0.11
Total dietary fiber content, %	61.22 ± 1.71
Insoluble dietary fiber content, %	13.45 ± 0.84
Ash content, %	2.07± 0.14

Values represent means of three replicated trials, ± standard deviation.

**Table 2:** Content of biologically active compounds and antioxidant activity in apple pomace powder.

Compounds	Mean Value ± SD
Total polyphenol content, mg GAE/g DW	6.03 ± 0.21
Total flavonoid content, mg QE/g DW	2.13 ± 0.13
Total tannins, mg TAE/g DW	0.61± 0.03
Total carotenoid content, mg/g DW	0.04 ± 0.01
Antioxidant activity (DPPH), µmol TE/g DW	22.82 ± 0.19

Values represent means of three replicated trials, ± standard deviation.

**Table 3:** The influence of the duration of ultrasound and microwave application on the equivalent weight, degree of methoxylation, galacturonic acid content, degree of esterification, total polyphenol content, antioxidant activity, and pectin yield in apple pomace extracts as a function of pH and hydromodule (the results are expressed as means ± standard deviations of three experiments).

Parameters	Hydromodule	Duration of Application, Min											
		Ultrasounds						Microwaves					
		15			30			5			10		
		pH 1.5	pH 2.0	pH 2.5	pH 1.5	pH 2.0	pH 2.5	pH 1.5	pH 2.0	pH 2.5	pH 1.5	pH 2.0	pH 2.5
Pectin yield (PY), %	1:10	2.74 ± 0.15 ^b^	1.50 ± 0.09 ^a,b^	0.98 ± 0.05 ^a^	7.04 ± 0.37 ^e^	2.03 ± 0.10 ^b^	1.68 ± 0.07 ^b^	10.71 ± 0.31 ^h^	4.49 ± 0.19 ^c,d^	1.18 ± 0.02 ^a^	16.94 ± 0.23 ^j^	10.17 ± 0.20 ^g^	4.50 ± 0.05 ^c,d^
	1:15	3.87 ± 0.32 ^c^	1.91 ± 0.08 ^b^	1.18 ± 0.06 ^a^	8.73 ± 0.48 ^f,g^	3.70 ± 0.29 ^c^	2.23 ± 0.15 ^b^	11.71 ± 0.29 ^h^	4.61 ± 0.14 ^c,d^	1.81 ± 0.05 ^b^	19.56 ± 0.06 ^k^	11.49 ± 0.16 ^h^	6.78 ± 0.08 ^e^
	1:20	4.20 ± 0.29 ^c^	2.06 ± 0.12 ^b^	1.39 ± 0.11 ^a^	9.91 ± 0.41 ^g^	4.30 ± 0.26 ^c,d^	2.88 ± 0.19 ^b,c^	13.86 ± 0.33 ^i^	5.26 ± 0.20 ^d^	2.28 ± 0.08 ^b^	19.88 ± 0.29 ^k^	13.54 ± 0.04 ^i^	6.37 ± 0.11 ^e^
Equivalent weight (EW), g/mol	1:10	768.1 ± 15.3 ^d^	1128.0 ± 27.8 ^f^	1799.2 ± 21.6 ^h,i^	401.7 ± 7.9 ^a^	613.1 ± 9.8 ^c^	907.7 ± 12.9 ^d,e^	803.7 ± 10.5 ^d^	1180.0 ± 18.2 ^f^	1879.3 ± 12.8 ^i^	526.3 ± 2.4 ^b^	739.6 ± 4.8 ^c^	1092.7 ± 11.3 ^f^
	1:15	652.1 ± 10.5 ^c^	1117.2 ± 29.6 ^f^	1927.1 ± 12.9 ^i^	401.9 ± 9.1 ^a^	559.6 ± 5.3 ^b^	900.0 ± 15.2 ^d,e^	773.7 ± 9.7 ^d^	1360.0 ± 10.0 ^g^	1947.9 ± 2.1 ^i^	490.6 ± 5.6 ^b^	663.0 ± 5.1 ^c^	1068.1 ± 10.4 ^f^
	1:20	652.5 ± 18.6 ^c^	1057.3 ± 11.5 ^e,f^	1659.0 ± 9.0 ^h^	378.3 ± 6.4 ^a^	546.8 ± 10.1 ^b^	849.1 ± 11.3 ^d^	594.8 ± 8.6 ^b,c^	1028.8 ± 12.4 ^e^	2261.7 ± 9.3 ^j^	421.6 ± 3.1 ^a^	596.4 ± 3.5 ^b^	979.9 ± 7.5 ^e^
Methoxyl content (MeO), %	1:10	5.47 ± 0.02 ^d^	6.40 ± 0.04 ^j^	6.67 ± 0.04 ^k,l^	5.14 ± 0.02 ^b,c^	6.13 ± 0.06 ^h^	6.63 ± 0.08 ^k,l^	5.02 ± 0.01 ^b^	6.17 ± 0.05 ^h,i^	6.24 ± 0.06 ^h,i^	5.01 ± 0.4 ^a,b^	5.87 ± 0.02 ^f^	6.20 ± 0.03 ^h,i^
	1:15	5.50 ± 0.03 ^d^	6.43 ± 0.06 ^j^	6.71 ± 0.07 ^k,l^	5.27 ± 0.02 ^c^	6.25 ± 0.04 ^i^	6.29 ± 0.05 ^i^	5.13 ± 0.03 ^b,c^	6.00 ± 0.04 ^g^	6.23 ± 0.05 ^h,i^	4.91 ± 0.03 ^a^	6.08 ± 0.04 ^g,h^	6.24 ± 0.03 ^i^
	1:20	5.55 ± 0.03 ^d^	6.38 ± 0.05 ^i,j^	6.81 ± 0.05 ^l,m^	5.05 ± 0.02 ^b^	6.30 ± 0.04 ^i^	6.63 ± 0.07 ^k,l^	5.04 ± 0.02 ^b^	6.07 ± 0.05 ^g,h^	6.39 ± 0.07 ^i,j^	4.88 ± 0.02 ^a^	5.75 ± 0.03 ^e,f^	6.25 ± 0.04 ^i^
Anhydrogalacturonic acid content (AUA), %	1:10	56.85 ± 0.34 ^e^	53.96 ± 0.41 ^d^	50.31 ± 0.28 ^c^	76.52 ± 0.58 ^m^	66.16 ± 0.37 ^i^	61.42 ± 0.31 ^g^	53.86 ± 0.26 ^d^	49.62 ± 0.26 ^b^	47.31 ± 0.18 ^a^	65.66 ± 0.28 ^i^	61.74 ± 0.46 ^g^	58.92 ± 0.30 ^f^
	1:15	59.45 ± 0.53 ^f^	54.47 ± 0.50 ^d^	49.16 ± 0.25 ^b^	77.18 ± 0.49 ^m,n^	68.25 ± 0.40 ^j^	62.37 ± 0.28 ^g,h^	55.25 ± 0.29 ^d,e^	48.94 ± 0.30 ^b^	48.17 ± 0.21 ^b^	71.19 ± 0.45 ^k^	64.59 ± 0.37 ^h^	57.55 ± 0.27 ^e,f^
	1:20	61.92 ± 0.51 ^g^	54.84 ± 0.47 ^d,e^	53.73 ± 0.35 ^d^	78.71 ± 0.67 ^n^	68.95 ± 0.43 ^j^	62.31 ± 0.31 ^g,h^	61.67 ± 0.32 ^g^	52.12 ± 0.25 ^c^	47.47 ± 0.20 ^a,b^	73.02 ± 0.47 ^l^	65.68 ± 0.30 ^i^	61.04 ± 0.31 ^g^
Degree of esterification (DE), %	1:10	59.87 ± 0.35 ^h^	64.71 ± 0.41 ^i^	72.91 ± 0.51 ^k^	38.16 ± 0.22 ^b^	51.43 ± 0.38 ^e^	56.18 ± 0.40 ^g^	49.86 ± 0.27 ^d,e^	62.55 ± 0.48 ^h,i^	71.37 ± 0.59 ^j,k^	43.76 ± 0.32 ^c^	53.11 ± 0.48 ^e,f^	55.55 ± 0.43 ^f,g^
	1:15	50.73 ± 0.29 ^e^	63.35 ± 0.38 ^i^	73.78 ± 0.47 ^k^	38.77 ± 0.24 ^b^	48.61 ± 0.29 ^d^	56.69 ± 0.43 ^g^	51.83 ± 0.35 ^e^	61.36 ± 0.32 ^h^	68.88 ± 0.41 ^j^	44.74 ± 0.38 ^c^	50.44 ± 0.37 ^e^	56.02 ± 0.52 ^f,g^
	1:20	48.54 ± 0.31 ^d^	63.29 ± 0.45 ^i^	73.50 ± 0.41 ^k^	36.47 ± 0.29 ^a^	48.30 ± 0.31 ^d^	54.67 ± 0.37 ^f^	46.40 ± 0.31 ^c,d^	59.16 ± 0.45 ^g,h^	71.02 ± 0.55 ^j,k^	38.69 ± 0.31 ^b^	47.74 ± 0.41 ^d^	52.45 ± 0.48 ^e,f^
Total polyphenol content (TPC), mg GAE/g DW	1:10	3.93 ± 0.04 ^c^	6.77 ± 0.07 ^f^	8.14 ± 0.06 ^g^	3.92 ± 0.05 ^c^	9.14 ± 0.11 ^h^	12.98 ± 0.18 ^k^	5.42 ± 0.08 ^d^	7.90 ± 0.09 ^g^	12.82 ± 0.21 ^k^	2.50 ± 0.02 ^a^	3.24 ± 0.07 ^b^	5.81 ± 0.04 ^e^
	1:15	4.10 ± 0.05 ^c^	12.26 ± 0.05 ^k^	12.07 ± 0.07 ^j^	4.56 ± 0.07 ^c^	6.54 ± 0.08 ^e,f^	12.59 ± 0.15 ^k^	2.28 ± 0.05 ^a^	7.12 ± 0.11 ^f^	10.56 ± 0.23 ^k^	3.27 ± 0.06 ^b^	6.02 ± 0.05 ^e^	5.98 ± 0.06 ^e^
	1:20	2.68 ± 0.08 ^a^	10.40 ± 0.08 ^i^	11.02 ± 0.10 ^i^	2.16 ± 0.04 ^a^	11.14 ± 0.09 ^i^	10.60 ± 0.11 ^i^	4.00 ± 0.03 ^c^	5.93 ± 0.07 ^e^	13.05 ± 0.07 ^k^	2.86 ± 0.04 ^b^	4.97 ± 0.06 ^d^	5.63 ± 0.04 ^d,e^
DPPH antioxidant activity (AA), μmol TE/g DW	1:10	6.96 ± 0.17 ^c^	8.43 ± 0.24 ^d^	12.76 ± 0.22 ^h^	6.15 ± 0.09 ^b^	12.14 ± 0.19 ^g^	18.32 ± 0.35 ^l^	11.66 ± 0.26 ^g^	12.65 ± 0.20 ^g,h^	13.91 ± 0.27 ^i^	10.43 ± 0.28 ^f^	10.71 ± 0.31 ^f^	11.41 ± 0.35 ^f,g^
	1:15	7.89 ± 0.20 ^d^	13.67 ± 0.31 ^h,i^	18.86 ± 0.41 ^m,l^	10.15 ± 0.14 ^f^	9.36 ± 0.07 ^e^	17.55 ± 0.31 ^k,l^	7.85 ± 0.16 ^d^	11.34 ± 0.24 ^f,g^	12.62 ± 0.21 ^g,h^	12.58 ± 0.19 ^g,h^	14.68 ± 0.37 ^i,j^	11.70 ± 0.30 ^g^
	1:20	5.53 ± 0.13 ^b^	11.35 ± 0.26 ^f,g^	15.76 ± 0.29 ^j^	4.32 ± 0.05 ^a^	13.44 ± 0.25 ^h^	15.94 ± 0.27 ^j^	10.70 ± 0.21 ^f^	9.95 ± 0.09 ^e,f^	16.39 ± 0.35 ^j,k^	11.33 ± 0.24 ^f,g^	13.36 ± 0.28 ^h^	11.47 ± 0.39 ^f,g^
DPPH antioxidant activity, %	1:10	13.37 ± 0.37 ^b^	14.82 ± 0.15 ^c^	22.18 ± 1.25 ^g,h^	11.17 ± 0.21 ^a^	21.88 ± 0.40 ^g,h^	30.75 ± 0.76 ^l,m^	21.68 ± 1.06 ^g,h^	23.17 ± 1.06 ^g,h^	24.44 ± 0.41 ^i^	22.28 ± 0.11 ^h^	25.57 ± 0.72 ^j^	18.21 ± 0.91 ^e,f^
	1:15	14.61 ± 0.35 ^b,c^	16.78 ± 0.61 ^d^	29.0 ± 0.41 ^k^	18.32 ± 0.86 ^e,f^	22.04 ± 0.29 ^g,h^	29.96 ± 0.24 ^l^	20.28 ± 1.21 ^f,g^	22.25 ± 0.57 ^g,h^	21.63 ± 1.02 ^f,g^	28.07 ± 0.07 ^k^	31.07 ± 0.40 ^l,m^	19.85 ± 0.43 ^f^
	1:20	14.98 ± 0.47 ^c^	15.67 ± 1.17 ^c,d^	22.15 ± 0.40 ^g,h^	14.86 ± 0.27 ^c^	23.68 ± 0.55 ^h,i^	29.85 ± 1.62 ^l,m^	20.11 ± 0.76 ^f,g^	23.21 ± 0.61 ^h,i^	22.78 ± 0.64 ^h^	22.68 ± 0.35 ^h^	28.78 ± 0.51 ^k^	19.57 ± 0.61 ^e,f^

The results are presented as the mean of three measurements ± standard deviation (SD). Different letters (^a–n^) designate statistically different results (*p* ≤ 0.05).

**Table 4:** Results of mutual analysis of the influence of pH (1.5, 2, 2.5) on pectin properties in all hydromodules (10, 15, 20 (*v/w*)).

Pectin Properties	Influence of pH on Pectin Properties (Bits)			
	Ultrasound-Assisted Extraction		Microwave-Assisted Extraction	
	15 min	30 min	5 min	10 min
Pectin yield	0.885	0.873	0.998	0.998
Equivalent weight (ME)	0.998	0.998	0.996	0.982
Methoxyl content (MeO)	0.958	0.836	0.755	0.755
Anhydrogalacturonic acid content (AUA)	0.836	0.985	0.821	0.645
Degree of esterification (DE)	0.995	0.996	0.996	0.591
Total polyphenol content (TPC)	0.491	0.812	0.916	0.522
Antioxidant activity (AA)	0.684	0.915	0.325	0.101

**Table 5:** Requirements for the sensory characteristics of fruit bars.

Sensory Characteristics	Description
Appearance, shape, and surface	Glossy surface, slightly sticky. Rectangular shape, susceptible to deformation
Consistency	Semi-hard
Color	Uniform color. A pronounced dark shade interspersed with pieces of dried yellow apples
Taste and smell	Sweet, typical for cherries and dried prunes, with a taste of dried apples and rosehips. No foreign tastes or smells have been identified

**Table 6:** Physicochemical parameters, total viable count, biologically active compounds, and the antioxidant activity of fruit bars during storage.

Parameters	Storage Period, Days				
	1st	90th	180th	270th	360th
Moisture content, %	30.0 ± 0.1 ^e^	28.5 ± 0.1 ^d^	26.4 ± 0.0 ^c^	25.1 ± 0.1 ^b^	23.6 ± 0.1 ^a^
pH	3.61 ± 0.03 ^a^	3.61 ± 0.02 a	3.64 ± 0.0 ^a^	3.75 ± 0.02 ^b^	3.95 ± 0.01 ^c^
Titratable acidity, % expressed in citric acid	1.12 ± 0.02 ^c^	1.08 ± 0.01 ^b^	1.05 ± 0.01 ^b^	0.84 ± 0.02 ^a^	0.83 ± 0.02 ^a^
Water activity (a_w_), c.u.	0.571 ± 0.002 ^d^	0.565 ± 0.003 ^d^	0.543 ± 0.001 ^c^	0.510 ± 0.002 ^b^	0.496 ± 0.001 ^a^
Total viable count (TVC), CFU/g	0 ± 0 ^a^	2.0 ± 0.1 ^b^	2.0 ± 0.1 ^b^	2.0 ± 0.1 ^b^	2.0 ± 0.1 ^b^
Total polyphenol content, mg GAE/g DW	7.68 ± 0.12 ^c^	7.63 ± 0.13 ^c^	7.57 ± 0.11 ^c^	6.24 ± 0.13 ^b^	5.59 ± 0.07 ^a^
Total flavonoid content, mg EQ/g DW	2.75 ± 0.05 ^d^	2.71 ± 0.09 ^d^	2.48 ± 0.02 ^c^	2.13 ± 0.04 ^b^	1.85 ± 0.05 ^a^
Inhibition DPPH, %	84.09 ± 1.33 ^d,e^	82.62 ± 1.35 ^d,e^	77.91 ± 0.48 ^c,d^	72.29 ± 0.39 ^b^	67.80 ± 0.56 ^a^
Antioxidant activity DPPH, µmol TE/g DW	24.85 ± 0.14 ^d^	24.80 ± 0.09 ^d^	23.52 ± 0.05 ^c^	22.31 ± 0.07 ^b^	20.14 ± 0.0 ^a^

The results are presented as the mean of three measurements ± standard deviation (SD). Different letters (^a–e^) designate statistically different results (*p* ≤ 0.05).

## Data Availability

No new data were created or analyzed in this study. Data sharing is not applicable to this article.
